# Seeking Windows of Opportunity to Shape Lifelong Immune Health: A Network-Based Strategy to Predict and Prioritize Markers of Early Life Immune Modulation

**DOI:** 10.3389/fimmu.2020.00644

**Published:** 2020-04-17

**Authors:** Jolanda H. M. van Bilsen, Remon Dulos, Mariël F. van Stee, Marie Y. Meima, Tanja Rouhani Rankouhi, Lotte Neergaard Jacobsen, Anne Staudt Kvistgaard, Jossie A. Garthoff, Léon M. J. Knippels, Karen Knipping, Geert F. Houben, Lars Verschuren, Marjolein Meijerink, Shaji Krishnan

**Affiliations:** ^1^Netherlands Organisation for Applied Scientific Research (TNO), Zeist, Netherlands; ^2^Arla Foods Ingredients, Aarhus, Denmark; ^3^Danone Food Safety Center, Utrecht, Netherlands; ^4^Danone Nutricia Research, Utrecht, Netherlands; ^5^Utrecht Institute of Pharmaceutical Sciences, Utrecht University, Utrecht, Netherlands

**Keywords:** biomarkers, immune networks, early life, machine learning, text mining

## Abstract

A healthy immune status is strongly conditioned during early life stages. Insights into the molecular drivers of early life immune development and function are prerequisite to identify strategies to enhance immune health. Even though several starting points for targeted immune modulation have been identified and are being developed into prophylactic or therapeutic approaches, there is no regulatory guidance on how to assess the risk and benefit balance of such interventions. Six early life immune causal networks, each compromising a different time period in early life (the 1st, 2nd, 3rd trimester of gestations, birth, newborn, and infant period), were generated. Thereto information was extracted and structured from early life literature using the automated text mining and machine learning tool: Integrated Network and Dynamical Reasoning Assembler (INDRA). The tool identified relevant entities (e.g., genes/proteins/metabolites/processes/diseases), extracted causal relationships among these entities, and assembled them into early life-immune causal networks. These causal early life immune networks were denoised using GeneMania, enriched with data from the gene-disease association database DisGeNET and Gene Ontology resource tools (GO/GO-SLIM), inferred missing relationships and added expert knowledge to generate information-dense early life immune networks. Analysis of the six early life immune networks by PageRank, not only confirmed the central role of the “commonly used immune markers” (e.g., chemokines, interleukins, *IFN, TNF, TGFB*, and other immune activation regulators (e.g., *CD55, FOXP3, GATA3, CD79A, C4BPA*), but also identified less obvious candidates (e.g., *CYP1A2, FOXK2, NELFCD, RENBP*). Comparison of the different early life periods resulted in the prediction of 11 key early life genes overlapping all early life periods (*TNF, IL6, IL10, CD4, FOXP3, IL4, NELFCD, CD79A, IL5, RENBP*, and *IFNG)*, and also genes that were only described in certain early life period(s). Concluding, here we describe a network-based approach that provides a science-based and systematical method to explore the functional development of the early life immune system through time. This systems approach aids the generation of a testing strategy for the safety and efficacy of early life immune modulation by predicting the key candidate markers during different phases of early life immune development.

## Introduction

The first 1,000 days of life is a period of growth and development in which the foundations of lifelong immune homeostasis and microbial colonization are established in humans (1). Alterations during this period, due to environmental and host factors, are considered to be potential determinants of health-outcomes later in life (2–4). Therefore, risk reduction measures or immune health interventions during these stages of life may be most effective and efficient for improving health, increasing quality of life, and lowering costs to society due to immune related diseases and disorders.

When developing immune health interventions in early life, the regulatory authorities (EFSA, JECFA) stress the need to address the safety of such interventions. However, currently there is no regulatory guidance about how to assess the risk and benefit balance of such interventions. At the moment final safety confirmation comes from expensive and lengthy clinical follow up studies using a set of guidelines (5–7). Therefore, a need for a science-based system approach to assess the safety and benefit of nutritional immune interventions, with a special focus on early life is clear. With such an approach animal testing can be reduced, refined or replaced.

Key to understanding the potential of early life immunity to shape lifelong immune health is the concept of ontogeny—the immune system development from fetal life through adulthood. Previously, our group made an inventory and compared the maturation of the immune systems of human, mouse, rat, and mini pig, based predominantly on existing (from literature) and newly generated histologic data (8). Critical time windows of immune organ development were identified in human and the above mentioned experimental species. However, less is known about the functional time frames of the developing immune system in humans. This knowledge is crucial to identify factors that need to be considered for assessing the safety and efficacy of early life nutritional interventions and exposure.

As the immune system is an enormously complex system, it is crucial to obtain more understanding about the biological structures and processes to be able to improve human (immune) health. However, due to the enormous wealth of information available, it is extremely difficult to obtain a complete picture of the biological basis of immune related diseases and health. Individual researchers are often restricted to so called “knowledge pockets” (9) covering only a small fraction of all available knowledge, and that fractional information is spread through literature or various databases. This fragmentation of information clearly hampers our understanding of the molecular processes underlying human health and disease. In order to obtain a complete picture, data integration from different sources is required.

Systems immunology combined with bioinformatics can provide sufficient knowledge to identify factors to assess the safety and efficacy of early life nutritional interventions and exposure (10–12). Recent technological advances permit collection and storage of large datasets at molecular and cellular levels (genes, gene products, metabolic intermediates, macromolecules, cells). So far, most studies or research groups collected data sets from several—omics-platforms to understand the larger (systems) picture by putting the pieces together, mostly through association networks (e.g., Protein-Protein Interaction network). Association networks are static and undirected networks. They provide lesser information than a directed causal network. However, creation of system-wide causal networks from omics data is a task that is largely tedious, and not pragmatic. This is because the amount of data spanning the molecular changes in spatio-temporal space is too large to capture the system knowledge within causal network in sufficient detail. Nevertheless, the dynamics of the immune system are better understood and characterized with the use of causal networks. Our intention here is to create causal networks of the early life immune system in a comprehensive and pragmatic manner.

Here, we generated causal immune networks in early life from literature sources that correspond to the 1st, 2nd, 3rd trimester of gestation (resp. EG, LG, MG), birth, newborn and infant period as part of a bioinformatics workflow, which also included subsequent network enrichment steps to generate comprehensive causal early life immune networks. The network-based approach developed here, enabled us to elucidate different phases of early life immune development in a systematical way to predict and prioritize biological functions and genes associated with immune functioning in early life. Moreover, this systems approach aids the development of a science-based testing strategy for assessing the safety and efficacy of early life immune modulation by predicting the key candidate markers during different phases of early life immune development.

## Materials and Methods

### Generation of the Basis of Early Life-Immune Networks Using Text Mining

**Figure 1:**
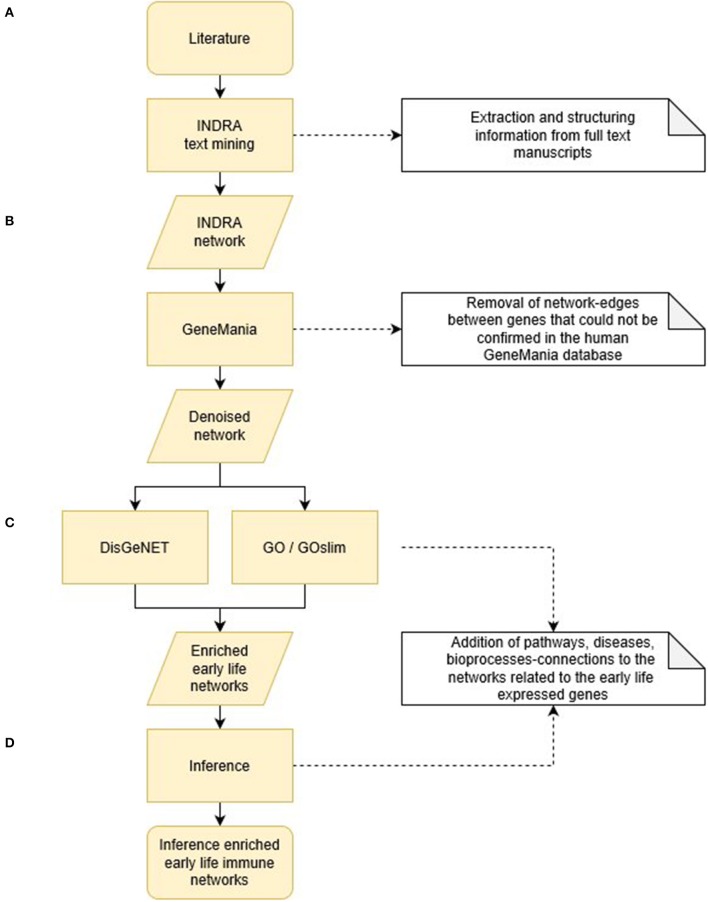
Bioinformatics workflow to generate human early life networks. **(A)** Expert based selection of early life immune manuscripts were divided in 6 early life time periods and subjected to INDRA text mining tool. This resulted in 6 causal INDRA network. **(B)** The gene-gene connections of the INDRA networks were denoised and validated for the human situation by GeneMania. **(C)** DisGeNET and Gene Ontology tools (GO and GOslim) enriched the denoised early life networks by adding gene-disease connections and gene-process/pathway connections. **(D)** Inference calculations enriched the early life networks further by adding process-disease and disease-immune health endpoint connections. All steps together resulted in 6 human early life immune networks. The results of the different programming steps are depicted in Tables 2–**4** as indicated.

**Figure 2:**
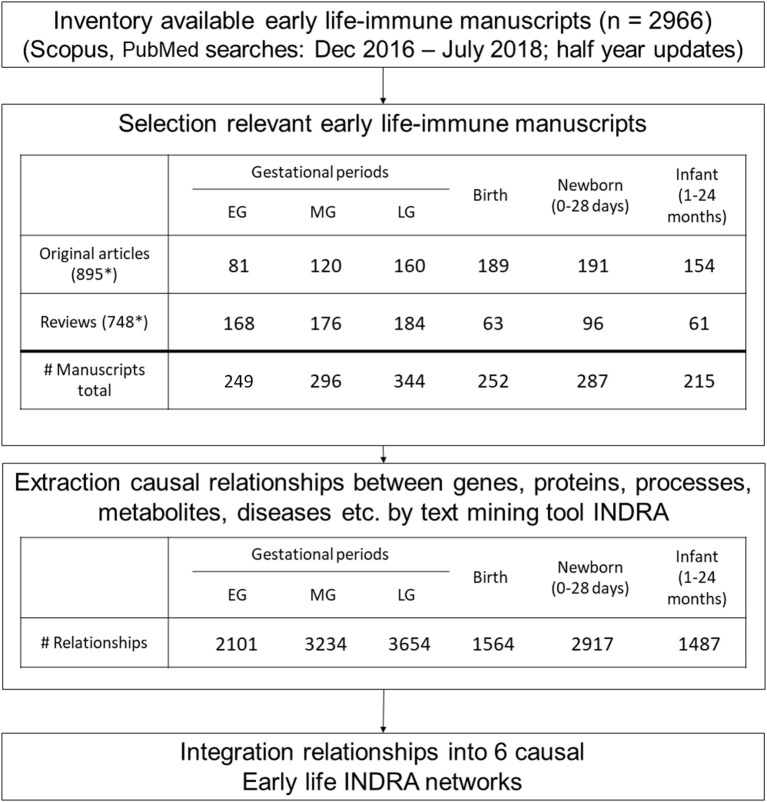
Workflow to generate the basis of early life immune networks by literature. Six causal early life immune networks covering a different early life were generated by selecting appropriate manuscripts from literature after which relationships between biological entities were extracted by the text mining tool INDRA. Next INDRA assembled, de-duplicated and standardized all relationships into causal early life-immune networks each covering a different early life period. These INDRA networks formed the basis of the early life immune networks. *Several unique articles cover multiple early life periods.

The entire bioinformatics workflow to generate human early life networks is depicted in Figure 1. The first step was to select relevant manuscripts describing immune mechanisms in early life. An inventory of the available literature regarding 6 immune developmental periods [1st/2nd/3rd trimester of gestation, birth, newborn (0–28 days), infant (1–24 months)] in human and experimental animals was made using Scopus and Medline (Figure 2). These databases were searched between 1st of December 2016 and 2nd of December 2016 and updated each half year (last update in March 2019). The search strings are depicted in Table 1. In total 2,966 articles were selected and manually screened on title, abstract and full text to select appropriate articles. Next, all selected articles were classified into the appropriate early life time period. The lengths of these different time periods in humans and experimental animals have been defined previously by Kuper et al. (8) and reported in Table 2.

**Table 1:** Search strings used to assess the available literature regarding the immune functional developmental stages in human and experimental animals was performed by searching the databases Scopus and Medline.

**Search terms**	**Combined with species terms**	**Combined with additional search terms**
Thymus OR spleen OR lymph nodes OR Peyer's patches OR bone marrow OR liver	Human OR mini pig OR rat OR mouse	•Functional AND developmental AND stages OR •Immune AND development AND birth OR •Immune AND development AND weaning OR •Immune AND development AND prenatal OR •Immune AND development AND postnatal
Cord blood	Human OR mini pig OR rat OR mouse	•Functional AND developmental AND stages OR •Immune AND development AND birth OR •Immune AND development AND prenatal
^*^	Human OR mini pig OR rat OR mouse	•Functional AND developmental AND stages AND (amniotic fluid) OR placenta OR (*in utero*) OR intrauterine OR •Immune AND development AND (amniotic fluid) OR placenta OR (*in utero*) OR intrauterine AND birth OR •Immune AND development AND (amniotic fluid) OR placenta OR (*in utero*) OR intrauterine AND prenatal

* *No additional organ/tissue-specific term used in this search string which is specifically aimed at the gestational phase*.

**Table 2:** Developmental early life stages in human, minipig, rat, and mouse [adapted from (8)].

**Early life period**	**EG^a^**	**MG**	**LG**	**Birth**	**Newborn**	**Infant**
Human	GD0–GW12	GW13–28	GW29–40	–	0–28 days	1–23 months
Minipig	GD0–GD37	GD38–75	GD76–113	–	0–15 days	2–4 weeks
Rat	GD0–6	GD7–13	GD14–21	–	0–7/10 days	1/1.5–3 weeks
Mouse	GD0–6	GD7–13	GD14–21	–	0–7/10 days	1/1.5–3 weeks

a *Starts at fertilization/conception*.

*EG/MG/LG, early/mid/late gestational period*.

*GD, gestational day; GW, gestational week*.

The text from the manuscripts was moderately preprocessed to correct for obvious noise in text that interfered with the text analyses. Noise correction included deletion of special characters (except numbers, letters, punctuations and hyphens), “Materials and Method” section, d.o.i., terms “fig.” and “table,” replacement of Greek characters by Roman letters, references containing “et al.,” and hyphenation if a word was split into two parts at the end of a line of text. The Python code used to preprocess the manuscripts can be found at https://github.com/TNO/immune_health_textmining/blob/master/PDFminer.py.

After this preprocessing step, INDRA (Integrated Network and Dynamical Reasoning Assembler) text mining platform (www.indra.bio/) was used to extract relationships and structure information on causal mechanisms among biological entities from the selected articles. INDRA is an automated model assembly system interfacing with NLP systems and ontology databases to collect knowledge, and through a process of network assembly, produce causal graph and dynamical models (13–15).

INDRA text mining platform rendered the full texts of the selected articles computationally accessible, identified biologically relevant entities (e.g., genes/proteins/metabolites/bioprocesses/diseases) and extracted relationships among these entities. Next, INDRA assembled, and standardized all relationships among the entities with associated evidence into causal early life-immune networks each covering a different early life period. Neo4J (https://Neo4j.com/) was used as a graph database management system to store, process and visualize the INDRA literature information as two-dimensional networks. This entire workflow is depicted in Figure 2.

Code used to generate the INDRA network is part of the INDRA repository and can be found at https://github.com/TNO/immune_health_textmining/blob/master/SRP_Neo4J.py.

### Denoising INDRA Literature Networks

In order to eliminate noise from the INDRA literature networks and only depict those relationships for which there is a biological indication that the relationship is valid, all gene-gene relationships in the INDRA literature network were subjected to a denoising step using GeneMania (https://genemania.org/).

Genes coding for proteins described in the INDRA network were entered in the GeneMania Cytoscape plugin (freely available at http://genemania.org/plugin/) to identify human gene-gene associations from its large collection of organism specific functional association data that include protein and genetic interactions, pathways, co-expression, co-localization, and protein domain similarity. These GeneMania-identified human gene-gene associations were compared to the gene-gene associations from the noisy INDRA literature networks, to identify and eliminate non-human specific associations between genes in the INDRA network. In the denoising step the edges (connections) between the genes were eliminated from the network, but not the genes themselves; they remained in the network as disconnected nodes. It must be noted that this step possibly eliminates true early-life gene-gene interactions if they are not represented in the human-specific GeneMania databases, which are mostly based on adult data. However, it is foreseen that this potential loss of information was compensated by the following enrichment steps because the disconnected genes remained part of the network. The code used to denoise the INDRA literature networks can be found at https://github.com/TNO/immune_health_textmining/blob/master/SRP_filter_networks.py.

### Network Enrichments (Figure 3)

The INDRA network derived from literature reflects only the functionalities of the genes and processes described in literature which provides an incomplete picture of the functionalities of the described genes because the manuscripts usually focus on a specific topic. Therefore, it was important to determine whether the expressed genes are associated with a certain biological process and/or molecular function and/or diseases which were not addressed in the selected manuscripts. This knowledge was retrieved from several databases and added to the networks (enrichment). To enrich the INDRA early life immune literature networks, the genes coding for the proteins in the network were entered into the Gene Disease Association Database (DisGeNET; http://www.disgenet.org/) to retrieve the gene-disease associations using WebGestalt tool (17). The same sets of genes were also entered in the Gene Ontology resource tools (GO enrichment tools GO and GO-SLIM; http://geneontology.org/) to retrieve gene-bioprocess associations (GO/GO-SLIM).

**Figure 3:**
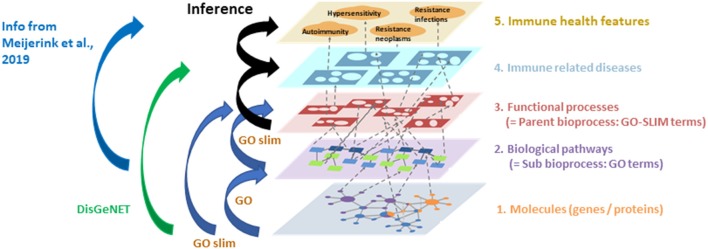
Overview of the steps used to enrich the INDRA networks. The genes described in early life literature (level 1). were entered in (i) DisGeNET to add gene-disease relationships to the network (level 1–4) and (ii) Gene Ontology tools GO/GO-SLIM to add gene-sub bioprocess (level 1–2), sub bioprocess—parent bioprocess (level 2–3) and gene-parent bioprocesses (level 1–3) relationships. Next the GO-terms linking to immune health features described previously in Meijerink et al. (16) were added to the network (level 2–5; blue arrow). The associations between bioprocesses and diseases (level 3–4) and disease–immune health features (level 4–5) were inferred (black arrows) based on the previous enrichment steps (orange arrows).

As a final step in the network enrichments, the associations among bioprocesses, immune related diseases and immune health endpoints (16) were inferred based on the enrichment tool specific database knowledge of the number and similarity of the genes related to each of the network entities in different layers in the model (Figure 3). As described earlier, Neo4J (https://Neo4j.com/) was used as a graph database management system to store and process all network information, including the literature-derived information by INDRA.

Codes used to generate these enriched networks can be found at https://github.com/TNO/immune_health_textmining/blob/master/SRP_Neo4J.py https://github.com/TNO/immune_health_textmining/blob/master/SRP_add_endpoints_to_disease_nodes.py and https://github.com/TNO/immune_health_textmining/blob/master/SRP_calc_inference.py.

### Prioritization Immune Markers in Early Life

In order to identify key early life genes (hub genes), the PageRank centrality score was calculated in the early life networks. The PageRank analysis was launched by Google (the web search engine) to identify significant web pages (18–20) and has been used for the analysis of networks in identifying the important nodes in the network (21). Unlike simply calculating the connections of each gene in the network, the PageRank score measures the importance or popularity of a gene based solely on the interaction (link) structure of the interaction network. It selects the genes that exhibit a high degree, whilst also maintaining the important low-degree genes, which link to other important genes in the network. The underlying assumption is that more important genes are likely to receive more associations from other important genes/bioprocesses/diseases.

The PageRank algorithm code can be found at https://github.com/TNO/immune_health_textmining/blob/master/SRP_calc_pagerank_neo4j.py.

## Results

### Generation of Early Life-Immune Literature Networks Using Text Mining

**Figure 4:**
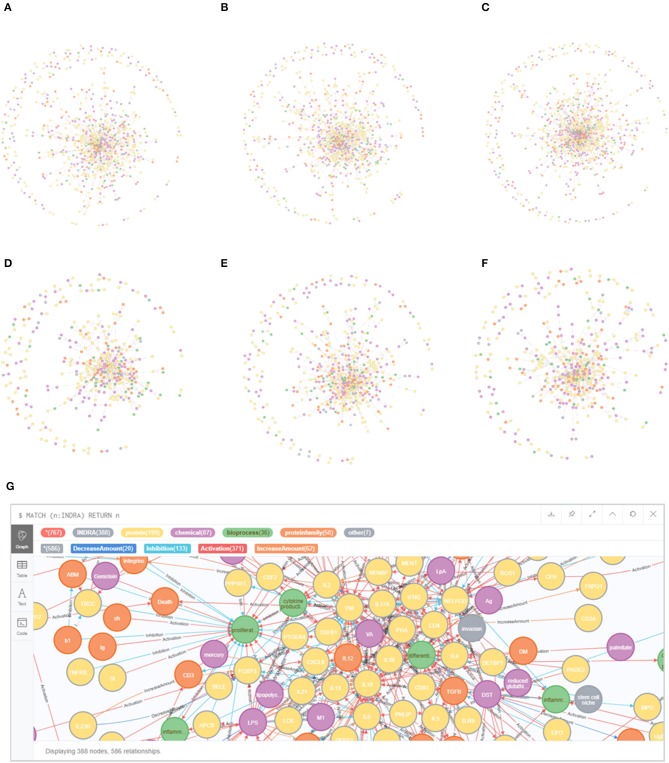
Early life immune networks based on information from early life immune literature and enriched with info from databases and inference steps, each covering a different phase during early life. **(A–C)** EG, MG, and LG; **(D)** birth; **(E)** newborn (0–28 days); **(F)** infant (1–24 months). **(G)** magnification of infant.

The literature covering the information on mechanisms involved in early life immune health is scattered across thousands of scientific papers. Therefore, text mining was applied to enable extracting and structuring information on causal mechanisms to create early life immune networks. In total 2,966 articles were selected using the search strings to explore literature databases. After manual screening 451 original manuscripts and 378 reviews were considered relevant (total number of selected 829 articles). This resulted in a selection of 249 articles for the 1st trimester of gestation, 296 articles for the 2nd trimester of gestation, 344 articles for the 3rd trimester of gestation, 252 articles for birth period, 287 articles for newborn period and 215 articles for the infant period. Please note that some articles covered multiple periods. From these full text articles, INDRA extracted resp. 2,101, 3,234, 3,654, 1,568, 2,917, and 1,487 unique relationships for the 1st, 2nd, 3rd trimester of gestation, birth, newborn and infant period (Figure 2). Next INDRA assembled, de-duplicated and standardized all relationships into 6 large early life-immune networks each covering a different early life period. The Neo4j-based framework enabled the visualization of the early life immune networks as depicted in Figure 4. As the networks are very dense in terms of numbers of nodes and edges, it is impossible to extract information directly from these networks without bioinformatical tools. The reason to depict these “unreadable” networks is to illustrate the complexity and density of them. In our methodology we identified 107 genes that have been described in the selected early life literature already during gestation and remained expressed throughout the infant period (Supplementary Figure 1).

### Denoising Early Life-Immune Literature Networks (Table 3)

Approximately 30% (range 27–32%, depending on early life period) of the connections (edges) between the genes coding for proteins described in the INDRA network were overlapping with the human gene-gene interactions present in the GeneMania consulted databases (Table 3), indicating that the denoising step reduced ~70% (depending on the early life network) of the gene-gene connections in our network. This large reduction may be due to the fact that: (a) The gene-gene connection is solely relevant in early-life situations, which are not reflected in the GeneMania-consulted databases (which contain mainly adult data); (b) The gene-gene connection is non-human specific as the search strings for literature included guinea pig, rat, and mice; (c) Only genes that could be linked to a unique HUGO Gene Nomenclature Committee (HGNC) ID are recognized by GeneMania; and (d) The gene-gene connection is nonsense and should therefore be excluded. It must be noted that only the edges between the genes are removed, but the genes themselves remained part of the network. Although this elimination step possibly also eliminates some of the true early-life gene-gene interactions as suggested above, it is foreseen that this potential loss of information was compensated by the following enrichment steps.

**Table 3:** Number of edges between genes described in early life (literature info) and their presence in the human GeneMania database.

**Network**	#Genes/proteins^*^ in early life literature extracted by text mining	**#Gene-gene edges in early life literature**	**#Edges confirmed in GeneMania (%)**
EG	440	228	72 (32%)
MG	477	278	84 (30%)
LG	508	319	90 (28%)
Birth	225	162	49 (30%)
Newborn	291	249	68 (27%)
Infant	232	174	51 (29%)

*EG/MG/LG, early/mid/late gestation*.

* *Sometimes it was not possible to distinguish protein names from corresponding gene names in literature. Therefore, all those names were annotated as being both a protein and a gene and regarded as 1 node in the network*.

### Network Enrichments

The relationships of genes coding for the proteins that were identified in the early life networks by text mining were enriched by information retrieved from Gene Ontology and DisGeNET databases, respectively, is depicted in Table 4. After enrichment, the number of gene—bioprocess relationships were increased 60-fold (approximately). Of note, depending on the early-life time frame, DisGeNET databases introduced numerous gene-disease relationships (ranging from 1,719 to 4,568 relationships) to the early life immune networks. Other than this, the DisGeNET database not being specific to immune-related diseases, numerous non-immune diseases were also added to the early-life immune networks.

**Table 4:** Results of enrichment/inference steps of the early life denoised INDRA immune networks.

	**#Gene-bioprocess edges**			**#Gene-disease edges**	**#Bioprocess-immune endpoint edges**	**#Bioprocess-diseases edges**	**#Disease—immune endpoint edges**
**Source**	**Literature**	**GO-enrichment**	**GO-SLIM enrichment**	**DisGeNET enrichment**	**Meijerink et al. (16)**	**Inference**	**Inference**
EG	149	9,546	443	3,894	1,121	1,701	1,023
MG	160	10,195	517	4,089	1,132	1,908	1,029
LG	180	10,968	546	4,568	1,246	2,207	1,136
Birth	67	3,929	168	1,719	695	1,073	627
Newborn	102	6,159	231	2,759	832	1,215	752
Infant	86	4,980	296	2,233	770	823	706

*Depicted are the number of connections (edges) between biological entities (genes, bioprocesses, diseases, immune endpoints) added to the INDRA immune networks. EG/MG/LG, early/mid/late gestation*.

Subsequent addition of associations between bioprocesses and immune health endpoints (autoimmunity, hypersensitivity, resistance to neoplasms, resistance to infections) as previously described (16), further enriched the early life immune networks. As a final step in the network enrichments, the connections between bioprocesses and immune related diseases and immune health endpoints were inferred based on the knowledge of the number and the similarity of genes shared among the entities in different layers of the model (Table 4 and Figure 3). The total number of nodes present in the early life immune networks after the enrichment and inference steps are depicted in Table 5, indicating the complexity of the resulting 6 human early life immune networks.

**Table 5:** Enriched early life immune network nodes.

**Type of nodes**	**EG**	**MG**	**LG**	**Birth**	**Newborn**	**Infant**
Proteins/genes^*^	440	477	508	225	291	232
Protein families	101	110	114	62	72	55
Chemicals	175	189	211	93	128	106
Bioprocesses^**^	51	56	58	36	39	34
GO processes	3,709	3,868	3,988	1,947	2,751	2,289
GOslim processes	55	55	55	59	60	59
Diseases	351	352	400	245	282	257
Immune health endpoint	4	4	4	4	4	4

*Depicted are the number of nodes in the networks after all enrichment/inference steps. These networks formed the basis of the gene prioritization (see Table 6). EG/MG/LG: early/mid/late gestation*.

* *Using text mining, it was not always possible to distinguish genes from proteins (often same name used)*.

** *Bioprocesses identified by ontology of INDRA text mining tool*.

### Gene Prioritization to Identify Key Markers in Early Life

The enriched complex human early life immune networks formed the basis to identify the key markers in early life. The PageRank score of all nodes was calculated in the 6 human early life immune networks which resulted in 6 lists of prioritized immune markers each covering a different early life period (Table 6).

**Table 6:**
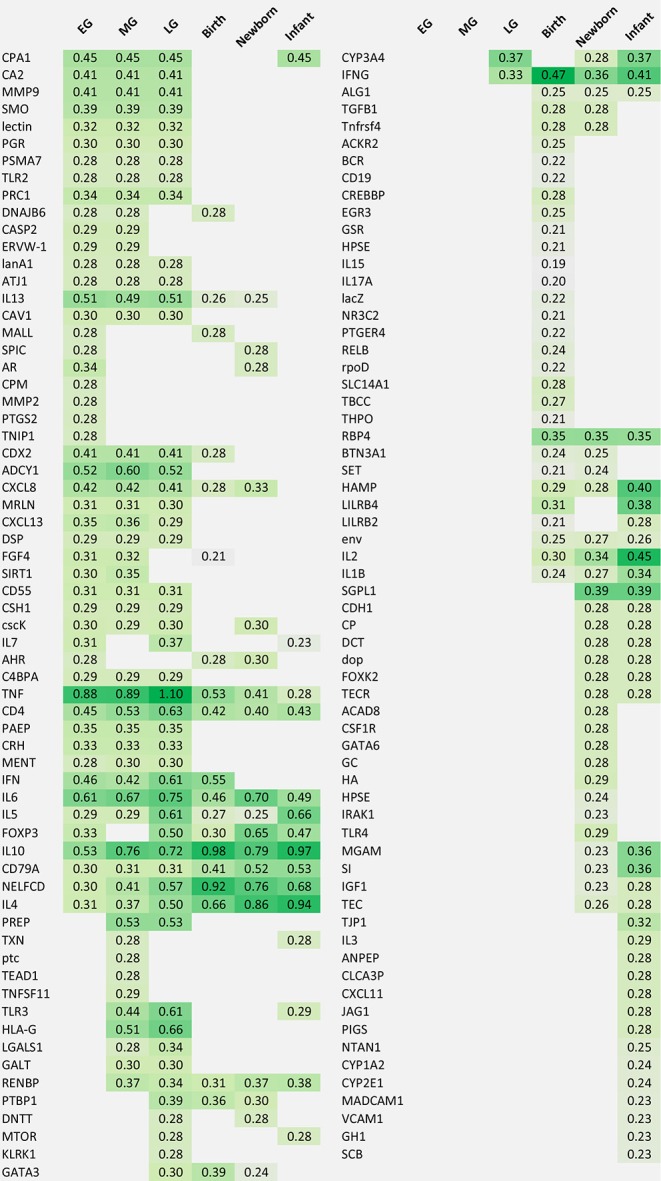
List of prioritized genes per early life time period.

*The PageRank score of all nodes was calculated for each gene in order to identify the most “central” genes in the networks. The top 50 genes (i.e., highest PageRank score) per network are depicted, including their PageRank score. EG/MG/LG, early/mid/late gestation. Descriptions of the genes are described in Supplementary Table 1. The light to dark green-gradient reflects the increase in PageRank score*.

In general, the genes coding for the “commonly used immune markers” were highly ranked in all early life periods such as the cytokines including chemokines (e.g., *CXCL8, CXCL11, CXCL13*), interferons (*IFN*), interleukins (*IL1B, IL2, IL4, IL5, IL6, IL7, IL10, IL13, IL15, IL17A)*, tumor necrosis factor (*TNF*), transforming growth *factor* (*TGFB*), and other immune activation regulators (e.g., *CD55, FOXP3, GATA3, CD79A, C4BPA*) directly involved in the immune response.

**Figure 5:**
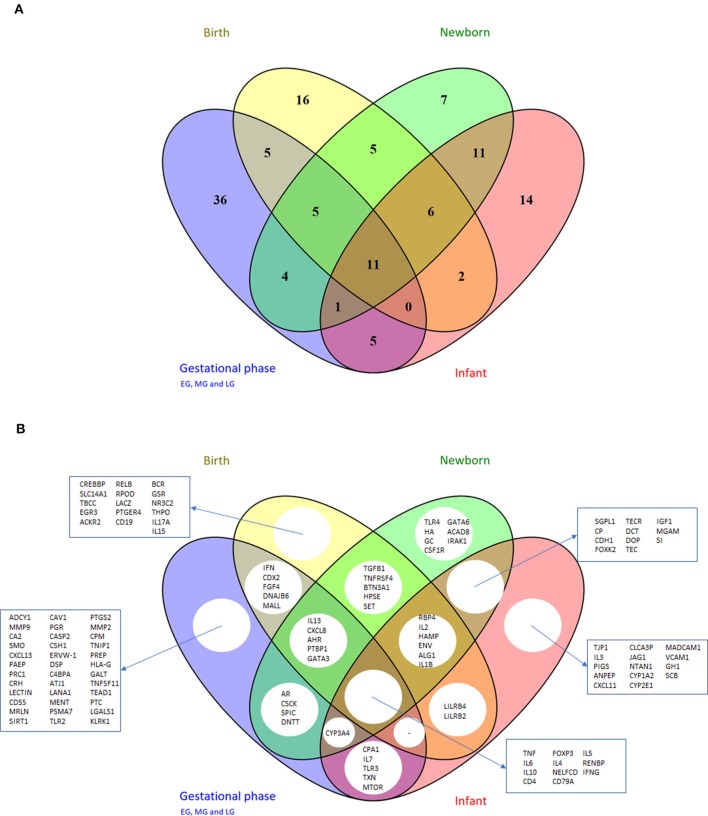
Venn diagram depicting unique and shared sets of genes from the top 50 gene lists of the different early life phases (Table 6); **(A)** number of genes and **(B)** gene names. For the gestational phases, the top 50 gene lists of early, mid and late period were combined, resulting in 67 unique genes. EG/MG/LG, early/mid/late gestation.

Comparison of the prioritized genes between the different early life periods (Figures 5A,B) showed that 36 genes were shown to be central in the network only during the gestational period, whereas others were more prominent in the periods birth, newborn and infant (6 genes: *RBP4, IL2, HAMP, env, ALG1*, and *IL1B*) or only in the infant period (14 genes: *TJP1, IL3, PIGS, ANPEP, CXCL11, CLCA3P, JAG1, NTAN1, CYYP1A2, CYP2E1, MADCAM1, VCAM1, GH1*, and *SCB*). Moreover, 11 genes were central in the early life immune networks covering all time periods: *TNF, IL6, IL10, CD4, FOXP3, IL4, NELFCD, CD79A, IL5, RENBP*, and *IFNG*. Most of these genes are immune related, however *RENBP*, renin binding protein, is an important regulator in the renin–angiotensin–aldosterone system. Moreover, *NELFCD*, Negative Elongation Factor Complex Member C/D, is an essential component of the NELF complex, which negatively regulates the elongation of transcription by RNA polymerase II.

Some of the top 50 genes were organ-specific such as *CPA1* (pancreas), *CRH* (neuronal), and *CDX2, MGAM, SI* (intestine). Other genes were specifically involved in pregnancy such as *ERVW-1, CSH1, PAEP*, or involved in early life growth, and maturation (e.g., bone/cartilage *CA2*, cell cycle related proteins *CAV1, PRC1*; matrix modulation *FGF4, MMP9, MMP2*) were also identified as central markers.

Interestingly, also a few non-human genes were selected in the top 50 lists (*lectin, cscK, lacZ, rpoD, dop, AtJ1, lanA1, env, ptc*), representing plant, bacterial or viral specific proteins as key markers. So although the GeneMania denoising step eliminated the gene-gene edges of non-human genes, these non-human genes got central positions in the enriched early life networks.

Concluding, the PageRank analyses resulted in the identification of key early life genes with overlapping genes between the different early life periods, but also genes which were only described in a certain early life period. Moreover, the PageRank analyses confirmed the central role of the “commonly used immune markers” (cytokines, chemokines) in the early life networks, but also identified less obvious key marker candidates.

## Discussion

In this paper, we describe an approach to construct early life immune networks to identify and prioritize factors to assess safety and efficacy of early life immune modulation. As an alternative to expensive, hand-built models which can take months to years to construct, a workflow was created to generate causal early life immune networks. Literature-based interactions were used to form the basis of the network. These literature networks were denoised using GeneMania databases and enriched with data from comprehensive databases, such as Gene Ontology and DisGeNET. Thereafter, PageRank algorithm was applied to prioritize candidate genes in the early life networks. The entire pipeline is interpretable and intervenable in a way that domain experts can use our tools to greatly reduce the time required to identify relevant immune markers in early life.

Early life in humans is associated with large developmental milestones in the immune system.

Innate and adaptive immune cells are present early in the fetus during gestation and then expand significantly (8, 22). However, though the innate and adaptive immune cells are already present early during fetal development in the first trimester of gestation, the strength of their effector functions differ considerably from the adult situation. For instance, mature neutrophils are moderately present at the end of the first trimester, and increase steeply in number shortly before birth. Their number then returns to a stable level within days, but they show weak bactericidal functions, poor responses to inflammatory stimuli, reduced adhesion to endothelial cells and diminished chemotaxis (23).

Compared with the adult immune system, which has matured and evolved after years of exposure to antigens and environmental stimuli, the newborn immune system comes from a relatively sterile environment and is then rapidly exposed to microbial challenges (10). It is well-established that these differences in exposure to antigens and environmental stimuli have consequences when examining disease susceptibility. Severe infections remain a leading cause of neonatal morbidity and mortality. The immaturity of the immune system is thought to be an important factor for the increased rate of neonatal infections especially when born preterm but the basis for this is not fully understood (12), although the maturation of the neutrophil and endothelial adhesion function are thought to contribute significantly to the high risk of life-threatening infections in premature infants (23).

Many of our preventive strategies for neonates rely upon our understanding of the adult immune system, because of our limited knowledge of early life immunity. Therefore, there is no consensus regarding which factors should be covered to evaluate the safety and/or efficacy of the early life interventions and how all the available data should be interpreted appropriately. Our bioinformatics approach assumes that the functions of genes and proteins do not change over time. Instead, the biological balances between gene-sets expressed in early life and adult are assumed to change e.g., lower FOXP3 and CTLA-4 expression in activated regulatory T cells from human neonates compared to the adult situation (24). Therefore, the enrichment steps using information from databases (GO and DisGeNET) containing mostly data from adult situations, are assumed to be suitable to enrich the networks with functionalities of the genes/proteins that are described in early life literature. As input for these databases, only genes shown to be expressed in a specific early life period were entered to exclude the possibility that genes/proteins that are not (yet) expressed in that specific time frame would be introduced in the network. As others, we suggest that not the gene function as such, but the context in which the genes are expressed in early life determines the impact of the gene expression on the biological processes, cellular responses and/or cellular phenotype of the immune cell. Especially the microbial context has been suggested to be important: the interactions between the developing immune system and the microbes colonizing the intestine, skin and airways of a newborn child has been suggested by several groups (11, 25, 26). Olin et al. (11) showed that the microbiome diversity increased after birth but children with exceptionally lower diversity indicating bacterial dysbiosis (and high level of activated T cell populations) showed an increased immunological heterogeneity at 3 months of age. Several key immune cell populations (DCs, B cells, NK cells), reach adult-like phenotypes during the first 3 months of life, which suggests that environmental exposures during this period could have influence later in life. For example, differential susceptibility to autoimmunity and asthma may relate to DC exposure to bacterial antigens early in life, which could lead to more tolerogenic DCs later in life (27–29).

Currently only a few biomarkers of inflammation have been developed into biomarker assays approved and recommended by regulatory bodies for use in clinical studies, which includes CRP, TNF-α, serotransferrin and erythrocyte sedimentation rate (30). Although many candidate markers are identified based on preclinical and clinical studies (as listed in the Thompson Reuters Integrity^SM^ Biomarkers Database), only a few are further validated and used for assay development highlighting the classical to clinical biomarkers gap. Moreover, in early life the identification of suitable markers is even more limited due to the fact that immunological studies on newborns tend to be small-scale and focus only on few factors because of limited sample volumes and low-throughput techniques as noted by Schaffert at al. (10). The early life immune networks generated in our approach enabled us to identify and rank genes that have the most central role in the early life immune networks. This is in contrast to earlier identified candidate markers for (pre-) clinical studies which are not specifically aimed at early life and not necessarily prioritized in a biological context.

There are multiple ways to prioritize genes in a biological network (31, 32). In computing network scores, most of the current approaches yield the limitation that the full network topology (systems approach) is not taken into account. Instead, such scoring methods focus on direct links or the most direct paths (shortest paths) within a constrained neighborhood around genes, ignoring potentially informative indirect paths. By applying PageRank algorithm, the full topology of the immune networks is taken into account.

Comparing the top 50 genes of the early life networks of the different time frames shows that many genes are already described in literature early in gestation. In general, the genes coding for the “commonly used immune markers” were highly ranked in all early life periods such as the cytokines including chemokines and other immune activation regulators directly involved in the immune response. Interestingly, transcription factors GATA-3 and FOXP3 that regulate Th2 and T regulatory cell development are highly ranked in the networks, whereas the gene coding for T-bet (*TBX21*), the transcription factor for Th1 differentiation, was in the lower regions of the priority lists. It has been shown that these 3 transcription factors cross regulate one another: T-bet modulates GATA-3 function and Th2 cytokines block Th1 differentiation (33–36). Additionally, GATA-3 has been shown to inhibit FOXP3 transcription by binding to the FOXP3 gene promoter (37). The low priority ranking of the gene coding for T-bet is in line with the current view of an unbalanced Th1/Th2 neonatal immunity resulting in skewing toward Th2 immunity. Moreover, the genes related to Th17 responses [transcription factor gene coding for RORγT (*RORC*) and *IL17A, IL17F*, and *IL22*], are also of low priority (not in top 50) in the networks. In the context of the neonatal Th2-biased immune response, the inhibitory effect of IL-4 on the development of inflammatory Th17-type responses has been described to represent a major regulation mechanism (38) which may explain the low priority of Th17 related genes and the high priority of IL-4 in the early life networks.

Several non-human genes (lanA1, cscK, dop, rpoD, lacZ, env, ptc, lectin) were ranked in the top 50, which might seem unexpected or perhaps even suggest a flaw in the bioinformatics approach. However, their presence and relevance may well-explained. In our workflow in the denoising step using GeneMania, we removed the connections between genes that were not of human origins, but we did not exclude the non-human genes from the early immune networks: the non-human genes remained in the network as disconnected nodes.

The next step in the generation of early life immune networks was the addition of connections (edges) between the human and non-human genes to human pathways/diseases/bioprocesses (input DisGeNet and GO databases). Genes from rat/mouse/guinea pig will likely not be connected to human processes, so these genes will stay disconnected to the network and therefore have a very low priority in the PageRank scoring. However, some of the non-human genes from mainly viral or microbial origin could be connected in our workflow to multiple human processes/diseases and therefore turned out to be in the top 50 of the PageRank scoring. The relevance of the role of these non-human genes in immune responses could be confirmed by literature: lanA1 (viral protein LanA1; role in host-virus interaction) (39), cscK (bacterial fructokinase; role in TLR4 activation) (40), dop (bacterial pup deamidase; role in resistance to infection) (41), rpoD (bacterial sigma factor for RNA polymerase; role in exponential growth bacteria) (42), lectin (role in activation of innate immune system) (43), lacZ (bacterial beta-galactosidase; Th1-associated) (44), env (viral envelope glycoprotein gp160; role in immune evasion) (45).

Several genes, which are usually not regarded as immune-related, got a prominent position in our early life immune networks such as genes involved in pregnancy, growth, and maturation (e.g., *ERVW-1, CSH1, PAEP, CA2, CAV1, PRC1, FGF4, MMP9, MMP2*). Several intestinal digestion related genes (*MGAM, ANPEP, SI*) were present in the top 50 in the birth-newborn-infant networks, which might be related to start of oral diet after birth. These examples emphasize the role of the immune system on so many other non-immune bioprocesses, which should be taken into account during assessment of possible (side-)effects of immune modulation in early life. Indeed, several chemokines and cytokines selected in our workflow, such as CXCL8, IL-10, TNF, IL1B, TGFb are multifunctional molecules initially described as having a role in endometrial functions and play a role in appropriate embryo implantation or placental functioning (46, 47). Moreover, TNF and TGFb have been identified as core activators of epithelial to mesenchymal transition, which is essential for embryonic development (48, 49). Although our approach to collect and structure and prioritize all available information from literature and databases to identify candidate markers is exhaustive, it also has its limitations due to the natural limitations in the curation process of the usage of enrichment tool-dependent auxiliary databases, and to inaccuracies derived from text mining. Others being annotation issues, such as the incomplete annotation of genes to GO terms and diseases (50, 51). Furthermore, the approach might be subjected to a reporting bias as it can be difficult to distinguish the absence of a gene in early life or a relationship between molecules/pathways from a lack of evaluation. In addition, we do not take the context of the gene expression into account whereas it is known that the context determines greatly the impact of the genes on biological processes, cellular responses and/or cellular phenotypes of the immune cells. Also, the networks are not organ-specific, although organ-specific genes are in the top 50 of prioritized genes, such as *CPA1* (pancreas), *CRH* (brain), and *CDX2, MGAM, SI* (intestines).

The strength/weight of the relationships in the network were not taken into consideration, but merely 6 association networks have been generated of possible biological relationships in early life immunity. The next important step for the applicability of this approach would be to validate these relationships based on gene expression data, which will guide us to validate the networks and moreover enable us to finetune the weighing of the various relationships in the network. This may result in a re-prioritization of the most important genes in a specific period in early life. Moreover, by using gene expression data, it becomes possible to identify critical time frames for specific immune modulation, because depending on the exposure, different pathways/processes may be activated. Even taking into account these current limitations, to the best of our knowledge, this is the first global overview of the early life immune system that can be used as a starting point to select putative markers to monitor the functioning of the early life immune system.

The future step would be to enrich the early life immune networks with early life gene-expression data to generate a quantitative early life immune network for (i) the analysis of mechanisms underlying immune health and disease in early life and (ii) the validation of candidate markers of disease and health.

In conclusion, we describe a network-based approach that provides a science-based and systematic method to explore the functional development of the early life immune system in time. This systems approach aids the generation of a testing strategy for assessing the safety and efficacy of early life immune modulation by predicting the key candidate markers during different phases of early life immune development.

## Data Availability Statement

All datasets generated for this study are included in the article/Supplementary Material.

## Author Contributions

JB, MM, LV, and SK contributed to the conception and design of the study. MM, MYM, and TR performed literature database searches and selection. RD, MS, and SK wrote scripts for the preprocessing of the manuscripts, GeneMania denoising, GO/DisGeNET database-searches and inference steps and PageRank algorithm score calculation. JB, MM, and SK wrote the manuscript. JB, RD, MS, MYM, TR, LJ, AK, JG, LK, KK, GH, LV, MM, and SK contributed to manuscript revision, read and approved the submitted version.

## Conflict of Interest

LN and AS are employed by Arla Foods Ingredients. JG is employed by Danone Food Safety Center. LK and KK are employed by Danone Nutricia Research. The authors declare that this study received funding from Arla Foods Ingredients and Danone Nutricia Research. The funders had the following involvement in the study: contributed to manuscript revision, read and approved the submitted version.

## Supplementary Material

Supplementary Figure 1 Venn diagram depicting unique and shared sets of genes of the different early life phases extracted from literature by INDRA text mining (Table 3) without PageRank prioritization step. EG/MG/LG, early/mid/late gestation.

Supplementary Table 1 Descriptions of the prioritized genes listed in Table 6.
