# Supplementary figures and images for: Seeking Windows of Opportunity to Shape Lifelong Immune Health: A Network-Based Strategy to Predict and Prioritize Markers of Early Life Immune Modulation
Source: Front Immunol. 2020 Apr 17;11:644. doi: 10.3389/fimmu.2020.00644 (PMC7182036; doi:10.3389/fimmu.2020.00644)

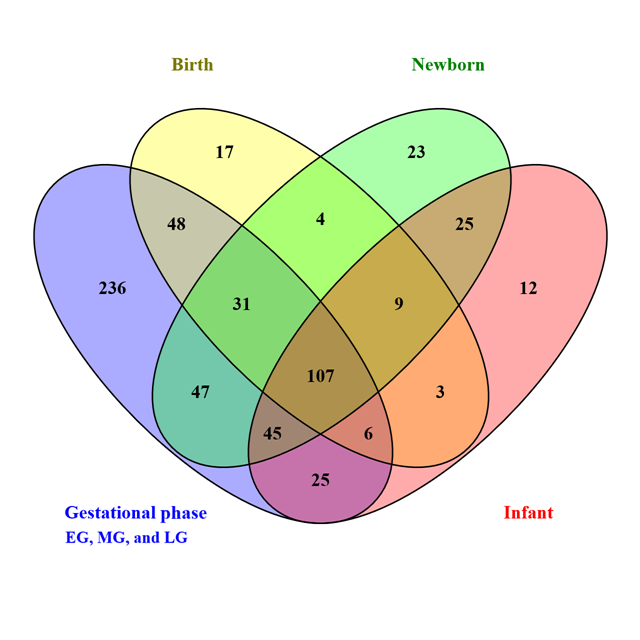

Supplement: Supplementary Figure 1 — Venn diagram depicting unique and shared sets of genes of the different early life phases extracted from literature by INDRA text mining (Table 3) without PageRank prioritization step. EG/MG/LG, early/mid/late gestation. [file Image_1.TIF]
